# Strategy Use in Second Language Vocabulary Learning and Its Relationships With the Breadth and Depth of Vocabulary Knowledge: A Structural Equation Modeling Study

**DOI:** 10.3389/fpsyg.2020.00752

**Published:** 2020-05-13

**Authors:** Na Fan

**Affiliations:** Department of English, School of International Studies, Shaanxi Normal University, Xi’an, China

**Keywords:** strategies, vocabulary learning strategies, vocabulary knowledge, structural equation modeling, individual differences

## Abstract

This study investigated Chinese English-as-a-foreign-language (EFL) learners’ use of vocabulary learning strategies (VLSs) and its relationship with vocabulary knowledge (VK), especially in relation to proficiency, gender, and discipline. Structural equation models were established following exploratory factor analysis (EFA) and confirmatory factor analysis (CFA) procedures, and mediation analyses and multiple-group analyses, as well as analyses of variance, were conducted. Four hundred nineteen sophomores’ strategy use frequency, Vocabulary Size Test (VST) scores (indicative of breadth of VK), Word Associates Test (WAT) scores (indicative of depth of VK), College English Test Band-4 scores, and gender and discipline categories were used as data. Proficiency significantly predicted Attention and Guessing positively but was a negative predictor of Socializing (asking others for help). Girls liked making notes while using dictionaries (DictNote) and Socializing, and students of arts also took more notes. Attention and Guessing significantly predicted VST and WAT positively, but Socializing significantly predicted the breadth and depth of VK negatively, and DictNote, Association, and Repetition had no significant relationship. The predictive power of Attention, Guessing, and Socializing, however, was achieved mainly, or for an important part, via the mediating or indirect effects of proficiency. Gender moderated the predictive power of Attention, Socializing, and DictNote over VST, greater for male students, whereas discipline moderated the relationship between Guessing and WAT, stronger for arts students. The findings are related to strategy features, gender characteristics, disciplinary influence, the EFL context and culture, and effective learning. This study reveals the complex relationships among use of VLSs, VK, and learner variables. Attention is called for to third-party factors in understanding VLSs–VK relationships. Given the important mediating effects of proficiency, it is proposed that vocabulary learning be strategically integrated into the accumulative process of English learning.

## Introduction

Scholarly efforts in the field of language learning strategies (LLSs) (see, e.g., O’Malley and Chamot, 1990; Oxford, 1990; Cohen, 1998) have been fundamental to the development of inventories of vocabulary learning strategies (VLSs). Using VLS inventories, researchers have explored a variety of issues, including what VLSs are adopted by successful and unsuccessful language learners (Gu and Johnson, 1996; Lessard-Clouston, 1996; Lawson and Hogben, 1998; Fan, 2003; Gu, 2003), what factors may influence the use of VLSs (Gu, 2002; Catalán, 2003), and how this use relates to breadth of vocabulary knowledge (VK) (Gu and Johnson, 1996; Kojic-Sabo and Lightbown, 1999) and to its depth (e.g., Nassaji, 2006; Zhang and Lu, 2015). Along these lines of inquiries, an updated investigation of use of VLSs among a different pool of Chinese participants may be warranted, given that the most well-known large-scale investigation of the use of VLSs in the Chinese English-as-a-foreign-language (EFL) context was conducted among Beijing Normal University students more than 15 years ago (i.e., Gu and Johnson, 1996; Gu, 2002), and considering that Chinese learners have been regarded as different from western ones (e.g., Watkins and Biggs, 1996; Gu, 2002). There may also be a need for statistical validation of the classifications of VLSs in several influential VLS questionnaires (e.g., Gu and Johnson, 1996) using factor analyses, so that with their inner structures tested, that reliance on the total or the average scores of items under certain or all categories as a measure of the use of VLSs, a practice many have criticized (e.g., Tseng et al., 2006), could be avoided, because initial items were examined and sifted and scores gained from individual items were weighted (e.g., Fabrigar et al., 1999; Zhang and Lu, 2015). Most importantly, the relationships between VLSs and breadth and depth of VK need further research. Zhang and Lu (2015) was the only study known to have employed structural equation modeling (SEM) to explore the VLSs–VK (the breadth and depth) relationships, based on Schmitt’s (1997) questionnaire. Different questionnaires can be used in eliciting data for SEM so that more categories of strategies can be included and studied. Also, the mediating and moderating effects of learner variables on such relationships have not been touched upon. The moderating effect concerns whether a relationship differs with individual differences, while the mediating effect determines if a third-party factor importantly but indirectly contributes to the relationship (e.g., Baron and Kenny, 1986; Mackinnon, 2011).

By addressing the above concerns, this study is also aimed to advance research on LLSs, echoing the consistent call for continued, but renewed, research in this field (e.g., Gao, 2007; Rose et al., 2018; Zhang et al., 2019). Rose et al. (2018) have commented Ardasheva’s (2016) and Teng and Zhang’s (2016) studies, both using SEM, “as having the greatest implications for driving forward the field of strategic learning” (p. 157). Similar to Ardasheva’s (2016) study, which explored the relationships between LLSs and reading and mathematics learning achievements, this study is concerned with the relationships between VLSs and vocabulary learning outcomes.

## Literature Review

### VLSs and Related Research

Vocabulary learning strategies refer to “a wide spectrum of strategies used as part of an on-going process of vocabulary learning” (Gu and Johnson, 1996, p. 669). In line with O’Malley and Chamot’s (1990) classifications of LLSs, Gu and Johnson (1996) developed an elaborate list of metacognitive and cognitive strategies that may be used from initial contact with words to putting them to use, which include categorizations such as selective attention, self-initiation, guessing, dictionary use, note-taking, memorization strategies, and activation. Schmitt (1997) and Fan (2003) have also constructed VLS questionnaires, based on O’Malley and Chamot’s (1990) and Oxford’s (1990) frameworks. The categorizations of VLSs and items built on theoretical grounds in these endeavors may need to be further validated with statistical methods such as exploratory factor analysis (EFA) and confirmatory factor analysis (CFA).

Research on VLSs started with examining the effectiveness of individual VLSs such as rote rehearsal (e.g., Prince, 1996), mnemonic strategies (e.g., Rodriguez and Sadoski, 2000), dictionary use (e.g., Fraser, 1999), and inferencing strategies (e.g., De Bot et al., 1997). Inferencing, for example, has been considered to be “a desirable strategy because it involves a deeper process that is likely to contribute to better comprehension of the text as a whole and may result in some learning of the lexical item that would not otherwise occur” (Read, 2000, p. 53).

Studies on use of VLSs (Gu and Johnson, 1996; Lawson and Hogben, 1998; Fan, 2003; Gu, 2003) have found that successful learners used a wider variety of VLSs in an orchestrated manner, with their own characteristics. Studies have also found that individual differences and learning contexts can exert influence on the choice and use of VLSs. Female students were reported to use more VLSs (e.g., Gu, 2002; Catalán, 2003) and to be more willing to try new ones (e.g., Young and Oxford, 1997) than male students. Arts students in China adopted significantly more note-taking strategies and spent more extracurricular time than did science students, whereas science students used significantly more word structure analysis strategies (Gu, 2002). With regard to learning contexts, Kojic-Sabo and Lightbown (1999), for instance, reported that English-as-a-second-language (ESL) learners employed more self-initiated VLSs, whereas EFL learners adopted more review strategies.

Then, studies that investigated the relationships between VLSs and learning outcomes have identified VLSs that may be conducive. Gu and Johnson (1996), for example, found that self-initiation, selective attention, guessing in contexts, skillful dictionary use, taking notes, attending to morphological features, encoding based on contexts, and attempts to use new words had very significant positive correlations with both vocabulary size and language proficiency (*r* from 0.14 to 0.35, all *p* < 0.001), whereas visual repetition of new words most strongly predicted both the two negatively (*r* = −0.23 and −0.24). Pitifully, in their study, the role of proficiency as a mediator in the VLSs–vocabulary size relationships was not further studied. In a similar vein, Kojic-Sabo and Lightbown (1999) reported that learners’ independence, time, dictionary use, and review positively correlated with word learning.

To my knowledge, only Nassaji (2006) and Zhang and Lu (2015) have explored the relationships between VLSs and depth of VK. Nassaji (2006) asked 21 intermediate ESL learners to read a text for comprehension and to try to infer the meanings of unknown words. Depth of VK was found to significantly relate to inferencing strategies and make a significant contribution to inferential success. Zhang and Lu (2015) analyzed the relationships between strategies and breadth and depth of VK using a sample of 150 university students. They found that strategies for learning word forms (e.g., spellings and sounds) and for studying meanings of words by establishing meaning associations (e.g., synonyms, roots, affixes, and other words in the semantic field) were significant and positive predictors of both vocabulary size (*p* = 0.002; *p* = 0.009) and VK depth (*p* = 0.007; *p* = 0.009), whereas using word lists negatively predicted breadth of VK (*p* = 0.021), both using word lists and using imagery (“Connect the new word to some situation in your mind” and “Make an image of the word’s meaning”) were negative predictors of depth of VK (*p* = 0.008; *p* = 0.001), and repetition was negatively but insignificantly related to both breadth and depth of VK (*p* = 0.915; *p* = 0.940). Noticeably, Nassaji’s (2006) study had a special interest in inferencing. Zhang and Lu (2015) adopted Schmitt’s (1997) questionnaire; their analyses focused on the consolidation strategies (i.e., strategies for consolidating learnt words), and their extracted factors did not include such well-known strategies as selective attention, note-taking, socializing, etc. Neither did they explore the moderating and mediating effects of learner variables in the relationships between VLSs and vocabulary learning outcomes. Additionally, both Gu and Johnson (1996) and Zhang and Lu (2015) included participants from the same university. There is a need for research with participants from different universities for modeling.

### Breadth and Depth of VK and Their Measurement

Vocabulary knowledge may consist of two equally important aspects: breadth and depth (e.g., Read, 1988, 1993, 1998; Wesche and Paribakht, 1996; Qian, 1999, 2002; Schmitt, 2010). Breadth refers to the number of words known by learners, or at least some significant aspects of the word meaning known by learners (e.g., Nation, 2001, 2006). Researchers have tried to measure the vocabulary size of L2 learners by developing vocabulary tests (Meara and Jones, 1988; Nation, 1990; Nation and Beglar, 2007). The Vocabulary Size Test (VST), developed by Nation and Beglar (2007), was selected for this study. It chose target words from BNC on a word family basis. Nation and Beglar (2007) suggested that word family count is preferable to lemma count for measuring receptive vocabulary size, because learners beyond a minimal proficiency level have some knowledge of word affixes and word building techniques. Then, VST covers 14 frequency levels, with each frequency level representing 1,000 words. The alternative answers were also designed to be closer in meaning so that testees’ VK could be best measured. It has displayed a high reliability (0.96–0.98) (Beglar, 2010).

Depth of VK concerns how well a learner knows a word (e.g., Read, 1993), and, taking a dimensional view, knowing a word means knowing all of its aspects (Richards, 1976; Nation, 1990, 2001). Nation (1990, 2001) proposed three facets of this knowledge: form, meaning, and use. Form embraces the spoken form, written form, and word parts; meaning incorporates form and meaning, concepts and referents, and associations; and use encompasses grammatical functions, collocations, and constraints on use (register, frequency, etc.). The Word Associates Test (WAT), developed by Read (1993, 1998), was adopted in this study, which measures: (1) word meaning, particularly polysemy and synonym, and (2) word collocations. The reliability of the test was reported to be 0.93 (Read, 1998), 0.91 (Qian, 1999), and 0.88 (Qian, 2002).

## Research Aims and Research Questions

The present study intends to offer a more recent overview of vocabulary strategy use in the EFL context and to provide more insights into VLSs–VK relationships, especially how the use and the relationships relate to learner variables. The research questions (RQs) are listed as follows:

(1) What are the VLSs that Chinese EFL learners use most and least frequently?
(2) Does use of VLSs differ with English proficiency, gender, and discipline?
(3) What are the relationships between the use of VLSs and the breadth and depth of VK? Does proficiency have any mediating effects, and does proficiency, gender, or discipline have any moderating effects on VLSs–VK relationships?

## Materials and Methods

### Participants

Participants were 419 Chinese non-English-major sophomores recruited from four universities in China; 243 of them (58%) were female and 176 (42%) male; 220 of them (52.51%) majored in arts, humanities, or social sciences (i.e., language and literature, education, economics, history, psychology, management, law, and media and journalism; hereafter, students of arts), whereas 199 (47.49%) majored in science and engineering (i.e., computer science, biology, civil engineering, chemical engineering, electrical engineering, and mechanical engineering; hereafter, students of science). Two of the universities were located in Chongqing in China’s southwest, one in Xi’an in northwest, and one in Kaifeng, Central China, and ranked around 30th to 80th among Chinese universities. While Gu and Johnson (1996) collected data from the most prestigious normal university in China, located in Beijing, and Zhang and Lu (2015) recruited participants from a comprehensive university in South China, these four universities were chosen, with considerations of access to participants, from a list of comprehensive universities in China’s Midwest, a less developed region in China that had not been focused on in investigations of this kind. Data from 36 students in the original pool of volunteers were excluded as they did not complete all the tasks. All participants reported their national College English Test Band 4 (CET-4) scores, ranging from 372 to 642 (52.39% to 90.42%, *M* = 484.02, *SD* = 51.01). For the needs of multiple-group analyses in SEM and analyses of between-groups variance, they were divided into high-, mid-, and low-proficiency groups based on *z*-scores (>0.5 for high, ≤0.5 but >−0.5 for mid, and ≤−0.5 for low), with 132, 140, and 146 of them belonging to each of the subgroups (in proficiency-related analyses, a misreport as an outlier was removed). The study was approved by the Australian Human Research Ethics Committees (HRECs) (ethics reference number: 5201300035). Table 1 is a crosstab showing the composition of participants.

**TABLE 1:** Participant composition.

Gender			Proficiency			Total
			High	Mid	Low	
Male	Discipline	Arts	7	15	45	67
		Science	42	46	21	109
	Total		49	61	66	176
Female	Discipline	Arts	39	48	66	153
		Science	44	31	14	89
	Total		83	79	80	242

*z-scores > 0.5 High, ≤ 0.5 but >−0.5 Mid, ≤ −0.5 Low; in proficiency-related analyses, an outlier was removed.*

### Instruments

#### VLS Questionnaire

The questionnaire adopted was primarily based on Nation’s (2001) VK framework (i.e., form, meaning, and use), and the majority of the items were adapted from Gu and Johnson’s (1996) as well as Schmitt’s (1997) VLS questionnaires. The formation of the questionnaire underwent piloting with 160 Chinese university students, modifications of translations and wordings, and adjustment of items. Expert advice assisted the choice of the framework, the adaptation of the questionnaire, and its language. The finalized questionnaire included 24 strategies for learning the meanings of new words, 28 strategies for learning how to use new words, and 5 metacognitive strategies that concerned learning both meanings and usages (see Appendix 1 for the English and Chinese versions), making it different from the two previous questionnaires because neither demonstrates a focus on, despite its coverage of, strategies for learning word usages. Technically, Gu and Johnson (1996) asked participants to ascertain the extent to which a statement was a true reflection of their vocabulary learning behavior, which might elicit their degrees of acceptance. To ensure collection of frequency data, I utilized a 5-point Likert scale eliciting frequency of use of vocabulary learning methods, which allowed five choices of “Never” (1 point), “Seldom” (2 points), “Sometimes” (3 points), “Often” (4 points), and “Usually” (5 points). I excluded form-learning strategies because Zhang and Lu (2015) have completed solid work there, and I was interested primarily in how meaning-learning and usage-oriented learning strategies may contribute to VK. The questionnaire I used was again validated via EFA and CFA procedures, which I will show in the analysis section.

The questionnaire required demographic data such as participants’ gender and discipline and CET-4 scores as proficiency data. The strategy items asked participants to tick their frequency of use. The Chinese version of the questionnaire was administered to prevent language barriers that might confound answers.

#### The Vocabulary Size Test

The VST (Nation and Beglar, 2007) contains 10 items for each of the 14 frequency levels of words, and the total score is 140. For each item, the testees are required to choose one answer that has a similar meaning to that of the target word. The bilingual version of the test was used because Nguyen and Nation (2011) found it as valid as the monolingual version, and excludes the confounding effects of English proficiency better. Below is an example of one item.

*They* ***saw*** *it*. (a) *cut*, (b) *waited for*, (c) *looked at*, (d) *started*

The reliability of the test in the present study was 0.70 based on Kuder–Richardson Formula 21 (K-R 21), which was acceptable as K-R 21 normally provides the conservative and minimum estimate (Alderson et al., 1995).

#### Word Associates Test

The WAT gives eight options contained in two boxes for each target word. All the target words are adjectives selected mostly from Barnard’s Second and Third Thousand Word Lists (Nation, 1986). An example of an item is shown below.


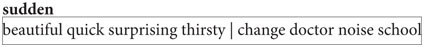


To complete the above item, test takers need to select four words related to *sudden*. The words in the left box (i.e., *quick* and *surprising*) may explain its meaning, whereas those words in the right box (i.e., *change* and *noise*) may collocate with it. The number of correct answers in either box is random. In this study, the reliability of the WAT reached 0.87 based on K-R 21.

### Procedure

The participants were asked to complete the VLS questionnaire, VST, and WAT in sequence in a session that was convenient to them. There was no time limit set as the tests aim to measure knowledge instead of fluency (Nation, 2012). The participants spent 75–115 min approximately on the survey and tests.

### Data Analysis

Data were analyzed with SPSS 23 and AMOS 23. First, descriptive statistics of strategy scores were obtained to identify the most and least used VLSs, to answer RQ1. Second, a succession of EFAs were conducted using the VLS scores of 210 randomly drawn participants, to extract latent factors and exclude items that did not fit within the categories. Multiple rounds of CFAs were conducted concomitantly, using the remaining 209 participants’ data, to validate the EFA results. The EFA and CFA procedures were performed also to enable weighted calculations of the frequency data in validated categorizations instead of simply averaging them in pre-assigned clusters. Third, SEM was administered to examine the relationship between use of VLSs and proficiency. Because categorical data, when used as independent variables, may not generate reliable relationships via SEM, *t*-tests were conducted to examine if strategy use differed with gender or discipline, using the scores of the factors as dependent variables, which were obtained via data imputation. Similar *t*-tests were also conducted to check if high- and low-proficiency groups differed in their strategy use. Step 3 was to answer RQ2. Fourth, a structural equation model was established to examine the relationships between use of VLSs and VST and WAT. Proficiency was then added to the model as a mediator, to estimate its indirect contribution to the relationships. Multiple-group analyses based on established SEMs were also conducted to examine whether the predictive power of VLSs over VST or WAT varied with proficiency, gender, or discipline, and how proficiency mediated this predictive power for separate proficiency, gender, and discipline subgroups. These steps were to answer RQ3. In all the above analyses, the normalities of the data were checked if necessary.

## Results

### Descriptive Statistics About Students’ Reported Use of VLSs

Of the 10 most used VLSs, three concerned dictionary use strategies (i.e., q 2.20, q 1.8, and q 1.6, *M* = 4.00, 3.74, and 3.71, respectively), four were related to guessing strategies (q 1.3, q 1.2, q 1.4, and q 1.5, *M* = 3.93, 3.79, 3.64, and 3.62), and two to repetition strategies (q 2.2 and q 2.1, *M* = 3.64 and 3.63). Additionally, the learners were often conscious of which new words were more useful and could learn them with selective effort (q 3.4, *M* = 3.98).

In contrast, memorization strategies such as making up stories (q 1.13, *M* = 1.61), writing words on papers and pasting them on the wall (q 1.23, *M* = 1.96), self-making a list of new words (q 1.12, *M* = 2.14), or relating words to senses (q 1.15, *M* = 2.42) were among the 10 least popular. So were using English–English dictionaries (q 1.7, *M* = 2.09) and copying example sentences from dictionaries (q 2.8, *M* = 2.48). New words were not often used, especially in speaking (q 2.15, q 2.17, and q 2.22, *M* = 2.38, 2.40, and 2.48). Finally, little attention was paid to how words were used while listening to English radio programs (q 2.11, *M* = 2.28).

### Factors Extracted From EFA and the Results of CFA

Five factors were extracted through EFA operations finally, and were named DictNote, Attention, Association, Guessing, and Socializing based on the 15 items they underlay. According to O’Malley and Chamot (1990) and Gu and Johnson (1996), DictNote, Association, and Guessing are cognitive strategies, Attention belongs to metacognitive strategies, while Socializing is a social strategy. The 15 items, their loadings, and the variance explained by each factor are given in Table 2.

**TABLE 2:** Factors extracted from EFAs.

Brief description of items	F1	F2	F3	F4	F5
2.7 Noting down collocations	0.905				
2.8 Noting down example sentences	0.802				
2.6 Noting down grammar information	0.797				
2.9 Noting down useful expressions	0.770				
2.13 Attending to word use when reading English novels		0.895			
2.14 Attending to word use when reading English newspapers		0.864			
2.12 Attending to word use when watching English movies or TV		0.720			
1.16 Creating imaginary contexts for memorization			0.833		
1.17 Relating new words to personal experiences			0.817		
1.15 Relating words to senses			0.738		
1.4 Guessing from real situations in life				0.811	
1.5 Guessing based on common sense and world knowledge				0.726	
1.3 Guessing from textual contexts				0.722	
1.11 Asking others about meanings					0.907
2.21 Asking others about word use					0.906
Variance explained in percentage (69.97)	25.81	13.05	11.52	10.41	9.18

*Extraction method: principal component analysis. Rotation method: Varimax with Kaiser normalization. F1 = DictNote; F2 = Attention; F3 = Association; F4 = Guessing; F5 = Socializing.*

The five-factor solution was validated through CFA procedures, yielding acceptable fit indices, as is shown in Table 3. The five factors were then used in further analyses.

**TABLE 3:** Commonly used fit indices of CFA.

	χ^2^/*df*	GFI	AGFI	CFI	TLI	IFI	NFI	RMSEA
Acceptable level	< 3	> 0.90	> 0.90	> 0.90	> 0.90	> 0.90	> 0.90	< 0.08
My model	1.235	0.943	0.916	0.983	0.979	0.984	0.920	0.034

### Use of VLSs by Learners of Different Proficiency, Gender, or Discipline

**FIGURE 1:**
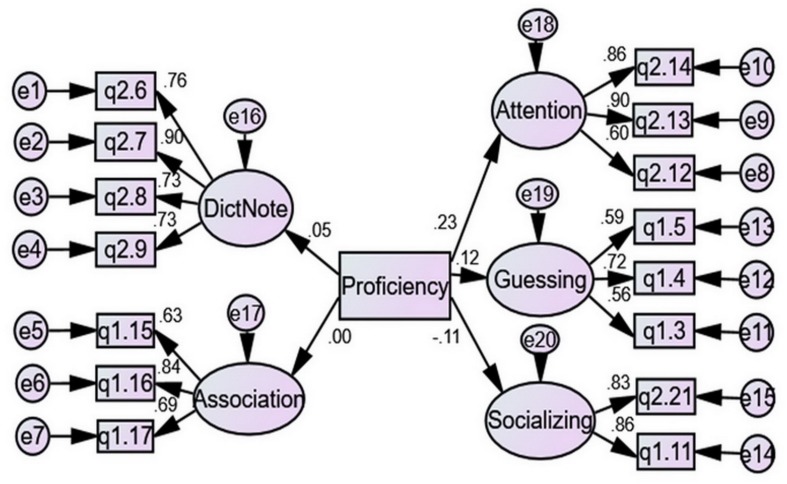
The structural equation model of the relationships between proficiency and use of VLSs.

Figure 1 shows the structural equation model of the relationships between proficiency and use of VLSs. Proficiency had significant positive predictive power over Attention and Guessing (*p* < 0.001 and *p* = 0.050, respectively), but significant negative predictive power over Socializing (*p* = 0.044). These indicate that as English proficiency increased, L2 learners attended more to word usages in their apparently extracurricular contact with English-learning materials, and engaged in more guessing or inferencing of meanings in textual or non-textual situations, but would less likely ask others for help. The results of *t*-tests showed that the high-proficiency group (*n* = 132, *M* = 2.658, *SD* = 0.842) significantly surpassed the low-proficiency group (*n* = 146, *M* = 2.298, *SD* = 0.832) in using Attention, *p* = 0.000, but not in employing other strategies.

Table 4 provides the model fit indices of the above SEM, all of which met acceptable levels, indicating that the regression coefficients in the model can be safely interpreted (Byrne, 2016).

**TABLE 4:** Model fit indices.

	χ^2^/*df*	GFI	AGFI	CFI	TLI	IFI	NFI	RMSEA
Acceptable level	< 3	> 0.90	> 0.90	> 0.90	> 0.90	> 0.90	> 0.90	< 0.08
My model	2.051	0.934	0.907	0.948	0.934	0.948	0.904	0.050

Significant gender and disciplinary differences were also revealed via *t*-tests. Females (*n* = 243) engaged in DictNote or took notes (*M* = 2.699, *SD* = 0.735) and asked others for help (*M* = 2.690, *SD* = 0.816) significantly more often than males (*n* = 176, *M* = 2.113, *SD* = 0.755, and *M* = 2.516, *SD* = 0.764, respectively), *p* = 0.000 and *p* = 0.027. Also, concerning DictNote, students of arts (*n* = 220, *M* = 2.619, *SD* = 0.773) significantly exceeded those of science (*n* = 199, *M* = 2.269, *SD* = 0.785), *p* = 0.000.

Given that repetition strategies (q 2.2 and q 2.1) were reported among the most frequently used, although the two items were excluded from SEM analyses for not being fit with any factor, the averages of their scores entered simple linear regression analyses and between-groups analyses. Results showed that the model containing only proficiency could explain 0.7% of the variation in the use of Repetition, indicating no significant relationship, *p* = 0.087. Also, no significant difference was found in use of Repetition between the high-proficiency group (*M* = 3.674, *SD* = 0.779) and the low-proficiency group (*M* = 3.517, *SD* = 0.846), *p* = 0.110, between males (*M* = 3.577, *SD* = 0.825) and females (*M* = 3.677, *SD* = 0.767), *p* = 0.201, and between students of arts (*M* = 3.593, *SD* = 0.806) and those of science (*M* = 3.681, *SD* = 0.777), *p* = 0.259.

### The Predictive Power of VLSs Over VK

**FIGURE 2:**
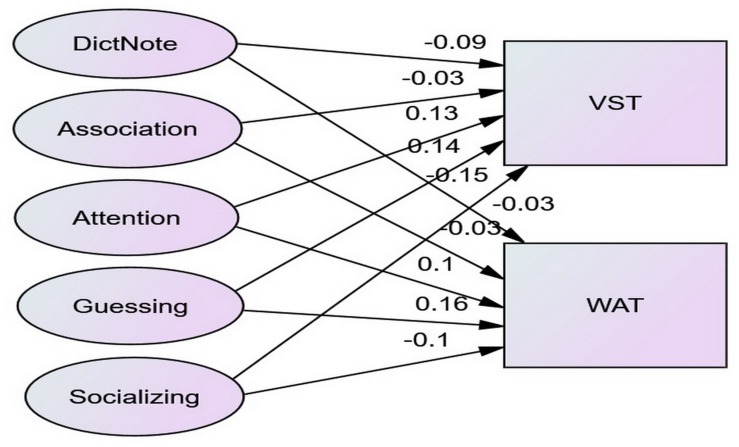
The structural equation model of VLSs, VST and WAT relationships.

Figure 2 presents the core of the structural equation model showing the extent to which the use of VLSs predicted VK. Again, all the eight model fit indices of the model met acceptable levels.

Table 5 presents the significant path coefficients in the above model. Attention and Guessing had significant or nearly significant positive predictive power over VST and WAT, whereas socializing had negative predictive power over the two.

**TABLE 5:** Significant path coefficients in the structural equation model.

	Attention	Guessing	Socializing
VST	0.13*	0.14*	−0.15**
WAT	0.10^a^	0.16*	−0.10^a^

*Significant at the 0.05 level. **Significant at the 0.01 level. ^*a*^Approaching significance, p = 0.07 and 0.06.

A simple linear regression was calculated to predict VST and WAT based on Repetition. No significant regression equation was found, with an *R*^2^ of 0.001 and 0.001, respectively, indicating no significant relationship between the use of repetition strategies and VST or WAT. Repetition negatively correlated with VST at −0.034, and WAT at −0.027.

### The Mediating Effects of Proficiency

**FIGURE 3:**
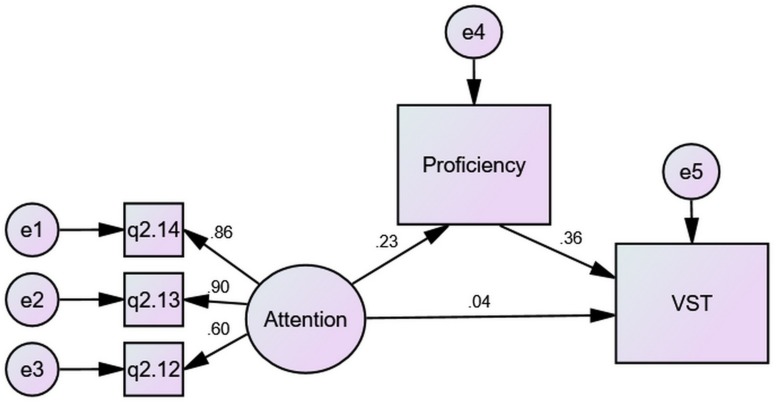
The mediating effect of proficiency compared to the direct effect.

The mediating effects of proficiency on the VLSs–VK relationships were also calculated via SEM. Figure 3 shows one such model where the indirect effect of Attention on VST through proficiency was 0.08 (0.23 × 0.36), greater than its direct effect, 0.04, making its total effects around 0.12. This indicates that while the predictive power of Attention on VST was significant, a larger part of it was realized via proficiency as a mediator.

Table 6 summarizes the direct and indirect effects of VLSs.

**TABLE 6:** The direct and indirect effects of VLSs on VST and WAT.

	Direct	Indirect
DictNote on VST/WAT	−0.06/0.00	0.02(0.05 × 0.37**)/ 0.02(0.05 × 0.37**)
Association on VST/WAT	0.02/0.03	0.00(0.00 × 0.37**)/0.00(0.00 × 0.37**)
Attention on VST/WAT	0.04/0.03	0.08(0.23** × 0.36**)/0.08(0.23** × 0.36**)
Guessing on VST/WAT	0.06/0.09	0.04(0.12* × 0.36**)/0.04(0.12* × 0.36**)
Socializing on VST/WAT	−0.10*/−0.04	−0.04(−0.11* × 0.36**)/−0.04(−0.11* × 0.37**)

Brackets include the predictive power of strategy use over proficiency × that of proficiency over VST or WAT. Proficiency correlated with VST at 0.365 and with WAT at 0.371, both p = 0.000. *Significant at the 0.05 level. **Significant at the 0.01 level.

As is shown in Table 6, all the direct effects were greater than the indirect effects (in absolute values) except for Attention, together with DictNote (on WAT), indicating that proficiency played a major role in the relationships between Attention and VST or WAT and between DictNote and WAT. However, given that the predictive power of Guessing or Socializing over proficiency was significant too, at 0.12 and −0.11, the role of proficiency was still of importance in their relationships with VK.

### The Moderating Effects of Proficiency, Gender, and Discipline

Multiple-group analyses established separate SEMs for subgroups and then compared regression weights pairwise to examine if they significantly differed. The results of multiple-group analyses are summarized in Table 7.

**TABLE 7:** Regression weights for subgroups in separate models in pairwise comparison.

	High/Low proficiency	Male/Female	Arts/Sci-tech
DictNote to VST	−0.04/−0.16	(−0.23**/−0.03)^a^	−0.05/−0.05
Association to VST	0.04/0.02	−0.01/0.02	−0.04/0.02
Attention to VST	0.07/0.11	(0.26**/0.04)*	0.04/0.19*
Guessing to VST	0.21/0.19^a^	0.19^a^/0.04	0.20*/0.05
Socializing to VST	−0.24**/−0.16	(−0.28**/−0.06)*	−0.16*/−0.17*
DictNote to WAT	−0.12/−0.07	−0.08/−0.04	0.04/−0.04
Association to WAT	−0.07/−0.06	−0.04/0.00	−0.05/0.03
Attention to WAT	0.15/0.11	0.07/0.13	0.05/0.12
Guessing to WAT	0.14/0.19^a^	0.17/0.14	(0.25**/−0.01)^a^
Socializing to WAT	−0.11/−0.03	−0.14/−0.08	−0.13^a^/−0.08

*Significant at the 0.05 level. **Significant at the 0.01 level. ^*a*^Approaching significance. () Significant in pairwise comparison.

The critical ratio (CR) for difference was calculated, which was the difference between the estimates of two regression loads, divided by an estimate of the standard error of the difference. None of the absolute values of the CRs for difference between high- and low-proficiency subgroups exceeded the benchmark value of 1.96, showing that the predictive power of the use of VLSs over VK for high-proficiency learners was not significantly different from that for low-proficiency learners.

As for the moderating effects of gender, Table 7 shows that, for male participants only, Attention significantly predicted VST positively, *p* = 0.00, as compared with female learners (CR = −2.16). The negative predictive power of Socializing over VST reached significance also for male learners, *p* < 0.001, but no such significant prediction could be made for females (CR = 2.45). A similar contrast was found for the relationship between DictNote and VST (CR = 1.73), which approached significance, where the negative predictive power was significant for male students only, *p* = 0.01.

**FIGURE 4:**
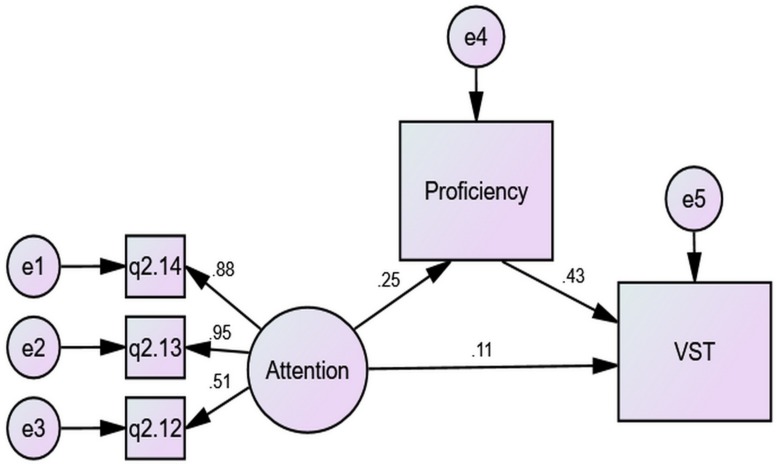
The mediation of proficiency for males.

Multiple-group analyses were further conducted to examine the mediating roles of proficiency for the respective gender groups. As an example, Figure 4 shows the mediation of proficiency in the Attention–VST relationship for male students.

**FIGURE 5:**
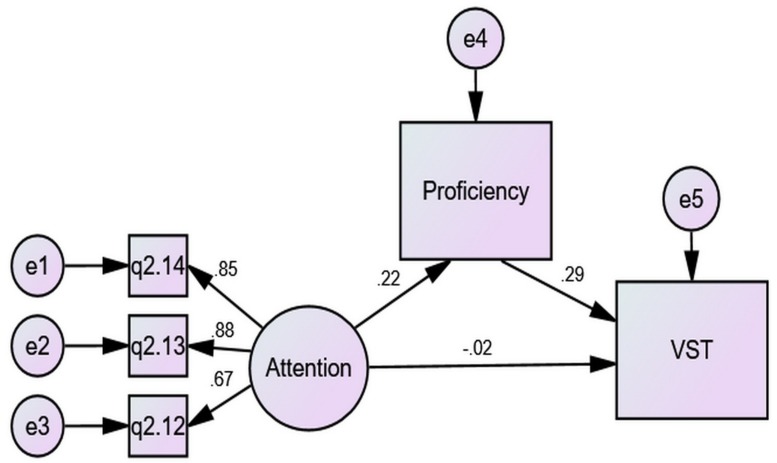
The mediation of proficiency for females.

In contrast, Figure 5 shows that mediation for female students.

A contrast of Figures 4, 5 unfolds that gender difference in the predictive power of Attention over VST derived from the difference in direct effects (0.11 vs. −0.02) as well as the difference in indirect effects (0.11 vs. 0.06), which arose mainly from the difference in the prediction of proficiency over VST (0.43 vs. 0.29). Male students gained more from Attention for their VST than females, and their proficiency was more closely related to vocabulary size.

Similar analyses also reveal that the predictive power of Socializing over VST for male students significantly surpassed that for female students due to differences in both direct effects (−0.17 vs. −0.04) and indirect effects (−0.09, i.e., −0.22 × 0.41 vs. −0.01, i.e., −0.04 × 0.28), the latter resulting from both difference in the predictive power of Socializing over proficiency (−0.22 vs. −0.04) and that of proficiency over VST (0.41 vs. 0.28). That is, when their proficiency and VST increased, male students no longer turned to others for help as much as before, whereas females would still engage in largely as much Socializing. The predictive power of DictNote over VST for male students surpassed that for female students significantly in direct effects (−0.19 vs. −0.01) instead of indirect effects via proficiency (−0.005, i.e., −0.01 × 0.45 vs. 0.01, i.e., 0.05 × 0.28), displaying a much greater negative relationship between frequency of DictNote and vocabulary size for male students.

Finally, no moderating effects of discipline were found, except that the difference in the predictive power of Guessing over WAT between arts and science students approached significance (CR = −1.83). Multiple-group analyses on the mediation model indicate that the predictive power of Guessing over WAT for arts students surpassed that for science students mainly in direct effects (0.17 vs. −0.01), instead of indirect effects via proficiency (0.06, i.e., 0.18 × 0.34 vs. 0.04, i.e., 0.12 × 0.33), showing that students of arts gained better from Guessing for WAT than those of science.

To summarize, Attention significantly predicted VST and almost significantly predicted WAT as well, positively, Guessing significantly predicted both VST and WAT positively, whereas Socializing significantly predicted the two negatively. Proficiency, which significantly correlated with Attention, Guessing, and Socializing, and with VST and WAT, made major or important contributions as a mediator to the predictive power of the three VLSs over VK. Neither DictNote nor Association nor Repetition significantly predicted VK. Both the Attention–VST relationship and the Socializing–VST relationship were significantly differential across gender, while the DictNote–VST relationship also differed, almost significantly, with gender, all being considerably stronger for male students. The Guessing–WAT relationship, however, was moderated by discipline, being stronger, almost significantly, for students of arts. These gender and disciplinary differences were attributable to differences in direct effects as well as indirect effects in the case of Attention and Socializing because of their particularly stronger relationships with proficiency for male students.

## Discussion

### Most and Least Used VLSs

An overview of the frequency data at the high and low ends helps understand current vocabulary learning behaviors in an EFL context. Using dictionaries and guessing have been found popular among Chinese students (Gu and Johnson, 1996; Fan, 2003) because EFL learners do not have a good mastery of English and meet new words frequently. They use English-Chinese dictionaries much more often because bilingual dictionaries offer word information in friendly local languages. In China, English majors are often required to use monolingual dictionaries, but the participants did not major in English. They also used both oral and written repetition strategies frequently. Chinese learners may take great effort for enhancing memorization, especially when learning a foreign language where tons of new words stay to be memorized. The importance of learning by repetition appears to have been emphasized in the Chinese culture. As an old saying goes, “Recite as many as 300 Tang poems, and you will be able to chant, even if you cannot write one.” As common classroom and homework practices, Chinese children read aloud and copy with a pen many times Chinese characters and words and, as they grow up, are required to recite recommended texts. The repetition strategies used for learning their first language may be transferred to learning English (see also Gu and Johnson, 1996). Interestingly, Schmitt (1997), Kojic-Sabo and Lightbown (1999), and Catalán (2003) also found that EFL learners tended to make use of dictionaries, employ inferencing (guessing) strategies, and engage in repetition.

Not surprisingly, some apparently “obsolete” or “cumbersome” ways of learning words (e.g., putting up new words on the wall) have been least used, as vocabulary learning resources become handy (electronized) and user-friendly. Listening to VOA or BBC on radio may still be recommended to English majors, although radio is out of favor. The learners do not use new words often, consistent with Gu and Johnson’s (1996) finding, as the EFL context affords few opportunities for daily use of English, hence the so-called “dumb English.” Mnemonics or situational association such as relating words to senses is rarely used because not many words can be related to senses.

### VLSs Used by Learners of Different Proficiency, Gender, or Discipline

Proficiency had significant positive predictive power over use of Attention and Guessing, but significant negative predictive power over use of Socializing. EFL learners of higher proficiency attended more to word usages in exposure to extracurricular learning materials of different genre, and there was significant difference between the high- and low-proficiency groups in employing this Attention. Higher achievers are more active in search for and better able to make use of extra learning resources, and reading English novels or newspapers or watching English movies or programs in itself sets a threshold of proficiency. Then, learners of higher proficiency engaged in more grounded guessing, or inferencing. This is consistent with Gu and Johnson’s (1996) finding that contextual guessing had positive correlations with proficiency, and one most successful group of Chinese EFL students, whom they called Readers, learned vocabulary through reading, inferencing, and contextual encoding mainly. Fan (2003) also found successful EFL and ESL Chinese learners frequently adopt inferencing strategies. They may be better able to infer meanings of words, which encourages them to continue to do so, because they have more resources, morphological, textual, situational, or cultural, to lean on, and can employ them more wisely. As for Socializing, it is natural that as proficiency improves, learners will become increasingly independent, hence the significant negative relationship. It is interesting that high- and low-proficiency learners did not differ significantly in the frequency of use of repetition strategies. The influence of learning by repetition may be prevalent, but learners may differ in the quality with which repetition strategies are utilized and in using a combination of other strategies.

Gender difference occurred only in DictNote and Socializing, with female students reporting significantly more use. This finding conforms to our common knowledge and observations. There seems to be a doctrine in China’s learning culture that attaches importance to note-taking for memorization, one version of which goes, “A poor pen is better than a good memory.” It appears to work on female students particularly. They tend to take better notes due to cognitive and motivational reasons, for example, conscientiousness (Reddington et al., 2015). It might also be assumed that, emotionally, for fear of forgetting or missing something important, female students are also more likely to take notes while consulting authoritative resources, which make them relieved and fulfilled. Their more frequent note-taking behavior might relate to social stereotypes as well, which assign roles such as secretaries or clerks to females. Socializing may be characteristic of female learners, too (e.g., Oxford, 1995). Psychologically and emotionally, female EFL learners are more inclined to ask their peers for help. It should be noted that the female and male participants did not differ significantly in proficiency (*n* = 242, *M* = 486.57, *SD* = 50.57, and *n* = 176, *M* = 480.53, *SD* = 51.55, respectively, *p* = 0.233); therefore, the effects of proficiency may be excluded in consideration of gender difference in strategy use.

Some studies in this strand (e.g., Oxford et al., 1988; Oxford and Nyikos, 1989; Wen and Johnson, 1997; Gu, 2002; Catalán, 2003) reported that female students used more strategies than did their male counterparts. Gu (2002) found that female students used significantly more VLSs relevant to the success of language or vocabulary learning, which included dictionary and note-taking strategies, as well as metacognitive strategies, contextual guessing, activation strategies, contextual encoding, and oral repetition. Catalán (2003) also reported that female students used significantly more formal rule strategies (e.g., analyzing parts of speech), input elicitation strategies (e.g., asking a teacher), and rehearsal strategies. While Gu’s (2002) and Catalán’s (2003) findings, put together, included dictionary use, note-taking, and socializing, one notable difference is that only DictNote and Socializing were identified in this study. This might be due to the use of the analytical tool, SEM, which made these gender differences salient, having the benefit of relying on categorizations of strategies validated via EFA and CFA procedures, which screened unstable items and allowed statistically tested analogous items to be clustered while giving them different weights. Another reason might be participant differences. In this study, female participants exceeded male participants only in VST (*M* = 66.29, *SD* = 9.37 and *M* = 64.14, *SD* = 11.81, respectively, *p* = 0.039), while in Gu’s (2002) study, the former outperformed the latter both in vocabulary size (*M* = 12.63, *SD* = 4.90 and *M* = 10.15, *SD* = 4.90, respectively) and general proficiency (*M* = 77.17, *SD* = 6.90 and *M* = 71.93, *SD* = 8.51, respectively), to a greater extent, both *p* < 0.001. Generally, female dominance did not appear as great in four comprehensive universities as in a specialized normal university reputed for her arts education.

In terms of disciplinary differences, students of arts used significantly more DictNote than students of science. This supports Oxford and Nyikos’s (1989) general report of disciplinary differences in choice of learning strategies and corroborates Gu’s (2002) finding. In Gu’s (2002) study, the most noticeable disciplinary difference lay in arts students using significantly more usage-oriented note-taking than science students (*M* = 4.45, *SD* = 1.14 and *M* = 4.12, *SD* = 1.16, respectively, *p* = 0.000). Since senior high school, Chinese students have opted for a disciplinary focus. Humanities and natural sciences differ in that the former represents a horizontal knowledge structure and a hierarchical knower structure containing parallel, often debatable knowledge claims made by the knowledgeable, while the latter represents a hierarchical knowledge structure and a horizontal knower structure resting on systematic generalizations achieved through scientifically recognized procedures (Bernstein, 1996, 1999; Maton, 2007; Cao and Hu, 2014). Students of arts may form the habit of taking notes because arts, humanities, and social sciences, prior to the sophomore level, may entail more imparting of different theories or schools of knowledge and memorization than calculation, application, and experimentation. As a general case at least in China, more girls major in humanities and social sciences. In this study, 153 out of the 220 students (69.55%) studying these disciplines were female. Gender preference to note-taking may also add to this disciplinary difference. Noticeably, the arts group differed significantly from the science group in proficiency (*n* = 220, *M* = 466.75, *SD* = 48.22, and *n* = 198, *M* = 503.22, *SD* = 47.11, respectively, *p* = 0.000). However, given that proficiency was not significantly related to use of DictNote, as is shown in Figure 1, I exclude the effects of proficiency in my discussion here.

### The Predictive Power of VLSs on VST and WAT

Attention to word usages in self-initiated exposure to English materials had significant positive predictive power over VST and could positively predict WAT almost significantly as well. Its predictive power over VST was especially distinct for male students, in both direct effects and indirect effects through proficiency. The fundamental roles of attention in second language learning have been well established (e.g., Schmidt, 1990). Gu and Johnson (1996) have also reported significant positive correlations between Selective Attention and vocabulary size and CET-2 scores (*R* = 0.24 and *R* = 0.26, both *p* < 0.001), and between Self-Initiation and the two outcomes (*R* = 0.35 and *R* = 0.30, both *p* < 0.001), with Self-Initiation being the strongest predictor among 20 categories of strategies, and Selective Attention the third. Why male students benefited more in VST from this Attention than female students remains unclear. It may be assumed that males might be better at or tend to focus better on learning vocabulary in this concomitant, analytical way, whereas female students might have concerns other than vocabulary learning, for example, plots or characters, while reading literary works or watching movies. This assumption needs to be tested, given the present lack of evidence in gender differences in second language incidental vocabulary learning. Interestingly, male students’ orientation to vocabulary learning in English learning may be evidenced by the much greater prediction of their proficiency over VST, which contributed to the gender difference in indirect effects, rendering us more confidence to claim that a good male Chinese sophomore English learner may have a large vocabulary than to judge a female learner similarly.

Guessing or inferencing could significantly, or nearly significantly, predict vocabulary size or depth of VK positively and could significantly predict proficiency as well. Its predictive power over WAT was especially distinct for arts students, who significantly surpassed science students in displaying direct effects. Many studies (e.g., Nation and Coady, 1988; Schouten-van Parreren, 1989; Hulstijn, 1992; Huckin and Bloch, 1993; Fraser, 1999) have found inferencing strategies effective for both reading comprehension and vocabulary learning. Inferencing involves learners’ hypothesis formation and testing about word meaning (e.g., Ellis, 1994), and such cognitive processing may result in better comprehension and vocabulary learning (Read, 2000). The presence of psychological and linguistic context of a text, as Schouten-van Parreren (1989) argued, could also facilitate learners’ memorization of new words. De Bot et al. (1997) further elaborated that inferencing involved gathering multiple sources of information, such as morphology, syntax, word associations, and derivations, a process in which learners may internalize word knowledge. The predictive power of Guessing found in this study was consistent with Gu and Johnson’s (1996) similar finding concerning the significant positive relationships between contextual guessing and vocabulary size, as well as proficiency. The finding also corroborated Nassaji’s (2006) study that showed a significant relationship between depth of VK and use of inferencing strategies. Although arts students did not have as much depth of VK as science students did (*M* = 96.30 and 100.79, respectively, *p* = 0.006), they gained more for their WAT from Guessing because, assumedly, their generally more training and engagement in extensive reading may enable them to better establish word associations and accept multiple facets of word meaning.

Socializing had significant negative predictive power over VST and a nearly significant negative relationship with WAT, with that over VST being significantly differential with gender, stronger for male students. Asking others for help is in itself caused by insufficiency in VK. Male students would significantly reduce their interpersonal consulting behaviors with increased size of vocabulary. In contrast, female students may employ the Socializing strategy as an opportunity to build rapport and to ask for confirmation, their intention to socialize unaffected as much even when their proficiency or vocabulary size increases.

DictNote did not have significant relationships with VK, except that it had significant negative predictive power over the VST of male students, which surpassed that for female students significantly in direct effects. Like Socializing, consultation of dictionaries or other sources and concomitant note-taking may suggest lack of VK. This is particularly the case with male students who do not seek for help as easily and are often surer of what they know. As male learners acquire more vocabulary and grow more independent, female students still appear to stick to such a strategy.

In addition, Repetition had no significant predictive power over both types of VK but negatively correlated with them. This is similar to Gu and Johnson’s (1996) finding concerning visual repetition and Zhang and Lu’s (2015) finding on repetition. As vocabulary size and proficiency grow, learners naturally reduce Repetition because Repetition is usually related to intensive memorization efforts. Besides, Repetition may be effective for short-term word retention (Hummel, 2010) and should be employed jointly with reviewing or other strategies to contribute to long-term acquisition of words. Association related to VK similarly, but it was much less often used than Repetition as a strategy (*M* = 2.58 vs. 3.63), probably because creating Association situationally with imaginary scenes, personal experiences, or senses does not appear efficient or widely applicable.

## Conclusion

This study finds that using dictionaries, guessing, and repetition are among the most used strategies in the present EFL context, but some other strategies have been least employed due to changes in time or lack of efficiency. Increased proficiency is significantly related to more use of Attention and Guessing and to less Socializing, girls engage in significantly more DictNote and asking others for help, and students of arts also take significantly more notes. These individual differences in using strategies are related to learner characteristics, strategy features, disciplinary structures, and the Chinese context and culture. As regards the VLSs–VK relationship, Attention and Guessing significantly or nearly significantly predict VST and WAT, positively, and Socializing significantly predicted the two, negatively, all with proficiency as an important mediator, indicating its critical role in understanding VLSs–outcomes relationships. Neither DictNote nor Repetition nor Association significantly predicts VK, but maintains a slightly negative relationship with it. Gender moderates the predictive power of Attention, Socializing, and DictNote over VST, all being considerably stronger for male students, whereas discipline moderates that of Guessing over WAT, interestingly greater for arts students. These moderating effects are attributable to differences in direct effects, as well as indirect effects via proficiency in the case of Attention and Socializing, and are discussed in light of gender differences and disciplinary influence.

Notably, this study has revealed the complex relationships among use of VLSs, VK, and proficiency, gender, or discipline, highlighting the important roles of these learner variables in mediating or moderating VLSs–VK relationships. A variety of interesting reasons that may underlie these relationships have also been considered. I conclude by calling for a more comprehensive understanding of use of VLSs and of the relationships between use of VLSs and VK, and more attention paid to the unique value of mediation and moderation analyses in second language relationship studies. Pedagogically, although relationship does not necessarily mean causality, it is suggested that vocabulary learning be strategically integrated into the prolonged, gradual process of English learning toward higher proficiency to increase VK as English proficiency increases, given the crucial role of proficiency as a mediator in the VLSs–VK relationship, and that care be taken for learners with different characteristics in the EFL context. It is also recommended that strategies identified as significantly predictive of long-term VK, such as Attention in self-initiated learning activities and situational Guessing, be particularly used in the process of learning vocabulary while learning English, but other strategies, such as repetition, association, socializing, or note-taking, as well as intensive learning efforts, admittedly, could play their due roles in necessary conditions. In fact, I see a promising combination of the strategies I find significantly predictive of VK with those interesting consolidation strategies reported by Zhang and Lu (2015).

This study has several limitations that leave room for further research. Admittedly, the questionnaire used is exploratory, and its original 57 items should be either reduced or expanded based on the EFA and CFA results for future use. For example, given criticisms that the quality of strategy use should be considered alongside the quantity (e.g., Dörnyei, 2005; Tseng et al., 2006), questionnaires may be developed or new items may be added that gauge learners’ quality of strategy use, or other complementary means, such as qualitative ones, as suggested by Rose et al. (2018) and Zhang et al. (2019), may be implemented. Also, the number of repetition items could be expanded so that the factor of Repetition, which was frequently used by the participants as a strategy category, might emerge in the factor analyses. Especially, the 15 items remaining in the analyses may be combined with the 15 items left in Zhang and Lu’s (2015) analyses for future survey use since all of them have been validated and represent different categories of strategies. It needs to be clarified, however, that questionnaire development was not the main aim here, and this limitation does not affect the results presented. Then, caution might be taken when the findings are to be applied to wider population, because of the constraints of the sampling method employed this study. Finally, it should be reminded that relationship should not be equated with causality, especially direct causality. Further studies may be warranted that examine the effects of VLSs on VK experimentally.

## Data Availability Statement

The datasets generated for this study are available on request to the corresponding author.

## Ethics Statement

The studies involving human participants were reviewed and approved by The Human Research Ethics team, Macquarie University. The patients/participants provided their written informed consent to participate in this study.

## Author Contributions

NF was the major designer and the sole implementer of the study. She interpreted the findings and wrote the manuscript.

## Conflict of Interest

The author declares that the research was conducted in the absence of any commercial or financial relationships that could be construed as a potential conflict of interest.
