# Supplementary material for: Strategy Use in Second Language Vocabulary Learning and Its Relationships With the Breadth and Depth of Vocabulary Knowledge: A Structural Equation Modeling Study
Source: Front Psychol. 2020 May 13;11:752. doi: 10.3389/fpsyg.2020.00752 (PMC7237738; doi:10.3389/fpsyg.2020.00752)
Supplement: Supplementary file 1 [file Table_1.DOCX]

Appendix 1 The vocabulary learning strategy questionnaire items

The English Version

Part 1: Strategies for learning the meanings of new words

| 1.1 I guess the meaning of a new word from its part of speech (e.g., whether it is a noun, a verb, an adjective, or an adverb). |
| --- |
| 1.2 I guess the meaning of a new word from its affixes and roots (e.g., *replay*, *re* means *do it again*). |
| 1.3 I guess the meaning of a new word from its textual context (e.g., surrounding words). |
| 1.4 I guess the meaning of a new word from real situations in daily life (e.g., from some traffic signs). |
| 1.5 I guess the meaning of a new word based on common sense and general information. |
| 1.6 I use an English-Chinese dictionary to learn the meaning of a new word. |
| 1.7 I use an English-English dictionary to learn the meaning of a new word. |
| 1.8 I pay attention to various meanings of a new word when I look it up in the dictionary. |
| 1.9 I take down the meanings of a new word when I look it up in the dictionary. |
| 1.10 I learn the meaning of a new word by comparing an English sentence with its translation (e.g., in bilingual advertisements or movie captions). |
| 1.11 When I don’t know the meaning of a new word, I ask others (e.g., my English teachers or friends) for its meaning. |
| 1.12 I make a list of new words to remember their meanings. |
| 1.13 I make up stories with new words to remember their meanings. |
| 1.14 I remember the meanings of new words based on semantic relations (e.g., synonyms, antonyms, etc.). |
| 1.15 I remember the meanings of new words by associating them with senses (e.g., sense of smell and hearing). |
| 1.16 I create imaginary contexts to remember the meanings of new words (To remember *scissor*, I imagine I need to cut something into pieces with scissors). |
| 1.17 I link a new word to my personal experience to remember its meaning (e.g., when I remember *happy*, I relate it to the happy things I’ve experienced). |
| 1.18 I remember the meaning of a new word in relation to the sentence in which the word is used. |
| 1.19 I remember the meaning of a new word in a phrase or an expression. |
| 1.20 I associate new words with real objects or physical actions to remember their meanings (e.g., when I learn the word *camera*, I think of a camera mentally). |
| 1.21 I use affixes and roots to remember the meanings of new words. |
| 1.22 I paraphrase or translate a new word to remember its meaning. |
| 1.23 I put up around my room small pieces of paper on which I write down new words and their meanings to remember them. |
| 1.24 I remember the meanings of new words by doing tests. |

Part 2: Strategies for learning how to use new words

| 2.1 I orally repeat a new word, its related phrase or the example sentence. |
| --- |
| 2.2 I write down several times a new word, its related phrase or the example sentence. |
| 2.3 I pay attention to its stylistic features (e.g., formality or being derogatory or complimentary) when I look a new word up in a dictionary. |
| 2.4 I pay attention to its grammatical information (e.g., countability) when I look a new word up in a dictionary. |
| 2.5 I pay attention to its collocations (i.e., its combinations with other words that happen very often, e.g., *fast food,* *The United States*, and *commit murder*) when I look a new word up in a dictionary. |
| 2.6 I note down the grammatical information of a new word when I look it up in the dictionary. |
| 2.7 I note down the collocations of a new word when I look it up in the dictionary. |
| 2.8 I note down the example sentences given for a new word when I look it up in the dictionary. |
| 2.9 I note down a useful expression or phrase when I see it. |
| 2.10 I pay attention to how words are used when I attend English lectures or presentations. |
| 2.11 I pay attention to how words are used when I listen to English radio programs. |
| 2.12 I pay attention to how words are used when I watch English movies or English TV programs. |
| 2.13 I pay attention to how words are used when I read English novels. |
| 2.14 I pay attention to how words are used when I read English newspapers. |
| 2.15 I try to use new words as much as possible in my speaking. |
| 2.16 I try to use new words as much as possible in my writing (e.g., in my diaries). |
| 2.17 I try to use different meanings of a new word in different contexts. |
| 2.18 I try to use the synonyms of a new word in my speaking or writing. |
| 2.19 I make up sentences using new words I just learned. |
| 2.20 When I don’t know how to use a word, I look it up in a dictionary. |
| 2.21 When I don’t know how to use a word, I ask others (e.g., my English teachers or friends). |
| 2.22 I try to use the collocations of a new word as much as possible in my speaking. |
| 2.23 I try to use the collocations of a new word as much as possible. |
| 2.24 When I use a word, I pay attention to its grammatical features. |
| 2.25 When I use a word, I pay attention to whether it is frequently used in the context. |
| 2.26 In using words, I choose formal or informal words according to contexts. |
| 2.27 I try to use a new word in an imaginary context. |
| 2.28 In using new words, I guess their collocations, that is, words frequently co-occurring with them. |

Part 3: Metacognitive strategies for learning both the meanings and usages

| 3.1 I learn only words that are related to exams. |
| --- |
| 3.2 I learn words from other channels besides textbooks (e.g., English novels and movies). |
| 3.3 I think it important and take effort to study usages of words as well as their meanings, spellings, and pronunciations. |
| 3.4 I think it more important and take more effort to study words that I think are more important. |
| 3.5 I make plans for studying English vocabulary (e.g., reviewing newly learnt words regularly). |

The Chinese Version

第一部分：学习新单词意思的方法

| 1.1 我会根据新单词的词性猜测新单词的意思（例如：根据这个单词是名词，动词，形容词或者副词来猜测它的意思）。 |
| --- |
| 1.2我会根据新单词的前缀或者后缀猜测新单词的意思 （例如：replay- 前缀 “re” 的意思是重新）。 |
| 1.3我会根据文章上下文猜测新单词的意思。 |
| 1.4我会从新单词出现的真实场景中猜测新单词的意思 （例如：从一些交通标志图片来猜测单词的意思）。 |
| 1.5我会根据自己的一些基本常识来猜测新单词的意思。 |
| 1.6我会用英汉词典学习新单词的意思。 |
| 1.7我会用英英词典学习新单词的意思。 |
| 1.8当我查词典时，我会注意词典中单词的各种不同意思。 |
| 1.9当我查词典时，我会记下词典中单词的各种不同意思。 |
| 1.10我会从中英文对照的句子中学习新单词的意思（例如：具有中文翻译的英文广告，具有中文字幕的英文电影等）。 |
| 1.11当我不知道单词意思的时候，我会向其他人询问单词的意思（例如：我的英语老师或者朋友）。 |
| 1.12我把新单词制作成单词表来记忆新单词意思。 |
| 1.13我会把新学到的单词编在一个故事里来记忆新单词意思。 |
| 1.14我会根据单词的语义关系来记忆新单词意思 （例如：同义词，反义词等)。 |
| 1.15我会用感官动作记忆新单词意思 （例如：把新单词和嗅觉，听觉等联系起来）。 |
| 1.16我会想象出一个可以使用新单词的场景来记忆新单词意思 （例如：当我记忆“剪刀”这个单词时，我会想象出一个我想要剪东西的场景）。 |
| 1.17我会把新单词和自身经历结合起来记忆单词意思（例如：当学习“happy” 这个词时，我会联想到以前一些快乐的事情）。 |
| 1.18我会把新单词连同它出现的句子一同记忆从而记忆新单词的意思。 |
| 1.19我会把新单词放在词组或者短语中来记忆新单词的意思。 |
| 1.20我会把新单词和现实事物或者肢体动作联系起来记忆新单词的意思（例如：当我学习 “camera” 这个词，我会想到照相机）。 |
| 1.21我会通过新单词的词根和词缀来记忆新单词意思。 |
| 1.22我会通过翻译或者用英文解释新单词的意思来记忆新单词意思。 |
| 1.23我会把写有新单词和新单词意思的纸条贴在房间里来记忆新单词意思。 |
| 1.24我会进行自我词汇测试来记忆新单词意思。 |

第二部分：学习新单词如何使用的方法

| 2.1 我会口头重复新单词，包含新单词的短语或者句子。 |
| --- |
| 2.2 我会重复书写新单词，包含新单词的短语或者句子。 |
| 2.3 当我查词典时，我会注意新单词的文体特征（例如：这个单词是否正式，是褒义词还是贬义词）。 |
| 2.4 当我查词典时，我会注意新单词的语法信息（例如：这个单词是可数名词还是不可数名词）。 |
| 2.5 当我查词典时，我会注意新单词的搭配（这个单词经常和哪些单词连用？例如：fast food; The United States; commit murder）。 |
| 2.6 当我查词典时，我会把新单词的语法信息记在笔记本上。 |
| 2.7 当我查词典时，我会把新单词的搭配记在笔记本上。 |
| 2.8 当我查词典时，我会把新单词的例句记在笔记本上。 |
| 2.9 当我看到一个有用的搭配或者词组时，我会把它记在笔记本上。 |
| 2.10 当我参加一些英文课程，英文报告时，我会注意单词的用法。 |
| 2.11 当我听英文广播时，我会注意单词的用法。 |
| 2.12 当我看英文电影或者英文电视节目时，我会注意单词的用法。 |
| 2.13 当我读英文小说时，我会注意单词的用法。 |
| 2.14 当我读英文报纸时，我会注意单词的用法。 |
| 2.15 我会尽量在口语中使用新单词。 |
| 2.16 我会尽量在写作中使用新单词 （例如：我的日记）。 |
| 2.17 我会在不同语境中使用新单词的不同意思。 |
| 2.18 我会尽量在口语或者写作中使用新单词的同义词。 |
| 2.19 我会用新单词造个句子。 |
| 2.20 当我不知道单词应该如何使用时，我会查词典。 |
| 2.21 当我不知道单词应该如何使用时，我会询问他人 （例如：我的英语老师或者朋友）。 |
| 2.22 我会在口语中尽量用到新单词的搭配。 |
| 2.23 我会在写作中尽量用到新单词的搭配。 |
| 2.24 当我使用一个单词时，我会注意它的语法信息。 |
| 2.25 当我使用一个单词时，我会注意它在这种语境中是否常用。 |
| 2.26 在单词使用中，我会根据不同语境选择正式或者非正式的单词。 |
| 2.27 我会想象出一个语境使用新单词。 |
| 2.28 我会在使用单词时猜测我认为经常和新单词搭配的一些单词。 |

第三部分：学习新单词意思、用法的元认知策略

| 3.1 我只学习和考试有关的单词。 |
| --- |
| 3.2 除了学习课本外，我还会通过其它途径学习英语单词 (例如：英文小说，英文电影)。 |
| 3.3 我注重学习单词的意思、拼写和发音，也会注重学习单词的用法。 |
| 3.4 我会更加重视学习我认为比较重要的单词。 |
| 3.5 我会制定计划学习英语单词（例如：定期复习新学到的单词）。 |
